# Improving Discharge Summaries on Vincent Square Eating Disorders Unit

**DOI:** 10.1192/bjo.2026.11514

**Published:** 2026-06-30

**Authors:** William Waugh

**Affiliations:** Central and North West London NHS Foundation Trust, London, United Kingdom

## Abstract

**Aims:**

Discharge summaries (aka electronic discharge notifications, eDNFs) are a vital part of care, ensuring clear and timely communication between services and allowing for an accurate record of care to be maintained. If eDNFs are not completed in a timely fashion, or not completed at all, service users struggle to access follow-up care, repeat prescriptions and referrals to other services. In mid 2024, on Vincent Square Eating Disorders Unit (VSEDS), eDNFs were not being completed in a timely manner – many were not completed at all. From May 2024 to August 2024, of 15 patients discharged:

- 5 had an eDNF completed at time of discharge (33.3%).
- 3 had an eDNF completed late (20%).
- 7 did not have an eDNF completed at all (46.7%).

Our aim: 70% of patients discharged from VSEDS will have an eDNF completed within 24 hours

**Methods:**

This project ran from mid 2024 to early 2025. We implemented 4 PDSA cycles, which included:

PDSA 1: weekly scheduled meetings between ward doctors and pharmacists.

PDSA 2: discharge medications prescribed and email sent to pharmacy at time that decision to discharge is made.

PDSA 3: attempted implementation of protected time for ward doctors to complete eDNFs (unsuccessful).

PDSA4: On Wednesday mornings pharmacy notified of all potential discharges; all discharge medications prescribed at this time.

Our QI project included input from a number of Experts by Experience, service users who have had previous experience of discharge from VSEDS. They emphasized the importance of a timely, clear and concise eDNF and spoke about the impact on their ongoing care and wellbeing. Some of their feedback is represented in our poster.

**Results:**

From November 2024 to February 2025:

- 14 patients were discharged from the ward.
- All of these patients had a discharge summary completed within 72 hours of discharge.
- 12 of these patients (85.7%) had a discharge summary completed within 24 hours of discharge.

**Conclusion:**

Our first PDSA cycle was unsuccessful – we found that in practice this simply added another meeting without making a difference to efficiency. PDSA cycle 2 appears to have the most impact by allowing pharmacy to process TTAs without having to wait for ward doctors to write a lengthy discharge notification first. We were ultimately unable to implement PDSA 3 successfully. In PDSA 4, we attempted to systematise PDSA 2 by setting a once weekly notification process for pharmacy regarding upcoming discharges.

